# Mechanical and Dynamic Mechanical Behavior of the Lignocellulosic Pine Needle Fiber-Reinforced SEBS Composites

**DOI:** 10.3390/polym15051225

**Published:** 2023-02-28

**Authors:** Dinesh, Bijender Kumar, Jaehwan Kim

**Affiliations:** Creative Research Center for Nanocellulose Future Composites, Department of Mechanical Engineering, Inha University, 100 Inha-ro, Michuhol-gu, Incheon 22212, Republic of Korea

**Keywords:** lignocellulose, pine needle fiber, thermoplastic elastomer, SEBS, fiber/matrix interface, mechanical properties, dynamic mechanical behavior

## Abstract

Aiming to generate wealth from waste and due to their significant fire threats to forests and their rich cellulose content, lignocellulosic pine needle fibers (PNFs) are utilized in this study as a reinforcement of the thermoplastic elastomer styrene ethylene butylene styrene (SEBS) matrix to create environmentally friendly and economical PNF/SEBS composites using a maleic anhydride-grafted SEBS compatibilizer. The chemical interaction in the composites studied by FTIR shows that strong ester bonds are formed between reinforcing PNF, the compatibilizer, and the SEBS polymer, leading to strong interfacial adhesion between the PNF and SEBS in the composites. This strong adhesion in the composite exhibits higher mechanical properties than the matrix polymer indicating a 1150 % higher modulus and a 50 % higher strength relative to the matrix. Further, the SEM pictures of the tensile-fractured samples of the composites validate this strong interface. Finally, the prepared composites show better dynamic mechanical behavior indicating higher storage and loss moduli and T_g_ than the matrix polymer suggesting their potential for engineering applications.

## 1. Introduction

Lignocellulosic natural plant fibers are being promoted as a replacement for traditional reinforcing fibers in polymer matrix composites due to their eco-friendly nature, renewability, ease of access, abundance, and low cost [1,2,3]. Natural fiber-reinforced polymer composites (NFRPCs) are attracting academic, research, and industrial interest because of their technological and economic benefits, such as simple and non-abrasive processing, light weight, good mechanical, physical, and thermal properties, low capital investment, and significant financial returns for farmers who grow these natural fibers [4,5]. NFRPCs have previously been studied, emphasizing their reinforcing fibers and the matrix polymers [5,6], their extrusion and processing [7], their properties and the influence of environmental parameters on their properties [8], their sound absorption behavior [9], their 3D printing [10,11], especially for biomedical applications [10], their recyclability [12], and their applications in construction, such as for ceilings, floors, partition panels, doors, windows, roof tiles, and furniture [13,14]; in transportation [14,15] for automobile door panels, car bumpers, glove boxes, floor panels, dashboards, and other units; and also in building and furniture, military, packaging, sports equipment, and medical applications [14].

Among various plants, with about 230 species and 11 genera [16], pine (Pinus) is the most abundantly available plant worldwide. Lignocellulosic pine needles are a major waste material falling from pine trees in large quantities yearly. They become extremely flammable after drying, catching fire in the forest, and resulting in severe losses for the local population. In addition to being significant fire threats, dried needles on the forest ground block the regeneration of seeds from the soil due to their slow degradation rate as they form a thick layer [17]. Pine needles have a low utilization rate and have not yet been fully employed for any industrial purpose, even though several uses have been recorded, including fiberboard, packing boxes, fiber extraction, lignosulfonate production, and pine wool production. Generally, the cellulosic and non-cellulosic components of the natural fiber determine the properties of the NFRPCs. With many advantages and properties, cellulose is the key component of natural fiber [18] that plays a major role in governing the mechanical properties of the composites. Red pine plant needles are rich in cellulose, containing 52–60% cellulose, 20% hemicellulose (80% holocellulose), 13–15% lignin, and 5% other extractives (pectin, wax, ash, etc.) with a tensile strength of 35.8–36.94 MPa [19,20]. The high cellulose contents of pine needles have the potential to be used as reinforcement to create polymer composite materials for better end products, thereby creating valuable wealth from waste. Very little research has been reported on pine needle fiber-reinforced polymer composites: pine needle fibers (PNFs) were utilized as reinforcement with polypropylene (PP) [17,20], polylactic acid (PLA) [21,22], epoxy [23], urea formaldehyde [24,25], phenol formaldehyde [26], resorcinol formaldehyde [27], and urea–resorcinol–formaldehyde [28] resin to develop their composites. Although, the use of PNFs as reinforcement for polymer composites and their effect on the dynamic mechanical behavior of the composites has not been extensively investigated. Thus, to create wealth out of waste, this study uses lignocellulosic PNFs as reinforcement from renewable resources to develop NFRPCs.

Due to their low processing costs, thermoplastic elastomers (TPEs) are used in various applications, including the subcomponents of automobiles, and now reinforced TPEs, or TPE composites, are replacing these TPEs in several applications [29]. Several TPEs are being used for engineering applications, and among them, styrene block copolymers are the most used TPE, comprising almost 50% of the TPE industry [30]. Styrene ethylene butylene styrene (SEBS) is an important TPE with high thermal stability, low modulus, and low temperature flexibility, which has been used in many industrial and commercial applications, including: plastic modification, adhesives, coatings, cables, wires, sealants, and medicals [31]. In addition, SEBS was also used as the polymer matrix for short fiber composites, such as alkali and N-alkylation-treated aramid fibers [32,33], polypyrrole-coated amorphous silica fibers [34], and also short natural fibers, including alkali and silane-treated pineapple leaf fibers [35,36] and alkali-treated banana fibers [37]. Maleic anhydride functionalized SEBS has recently been used as a matrix polymer with untreated bagasse fibers to develop its composite with improved mechanical properties by producing an interface between the untreated fibers and the functionalized matrix [4]. Thus, with a view to substituting TPEs for automobile applications with reinforced TPEs, a SEBS thermoplastic elastomer is chosen for the NFRPC being developed in this research.

For a composite material, in addition to the properties of the reinforcing fiber and the matrix, the fiber/matrix interface also plays a significant role in determining the final mechanical performance of the resultant composite [38]. Thus, the fiber/matrix interfacial adhesion is a major factor in determining the response of a composite to an applied load and its integrity under stress governing the properties of that composite. Lignocellulosic plant fibers that are hydrophilic and polar do not adhere well to hydrophobic and non-polar polymer matrices in their composites; consequently, the mechanical performance of the composites is adversely affected. An efficient way to improve the mechanical characteristics of the incompatible phases is to use compatibilizers or coupling agents that join the natural fiber reinforcement and the matrix polymer in the composite [39]. The compatibilizers are typically functionalized polymers which have a matrix-compatible backbone and functional groups attached to this backbone that can interact with the reinforcement [40]. Several coupling agents and their functions as compatibilizers in composites made of natural fibers and polymers were investigated [40,41]. Maleic anhydride-grafted SEBS (MAH-SEBS) has a great advantage in being used as a compatibilizer with the SEBS matrix, as it has an identical polymer backbone that can easily be miscible with the SEBS polymer, and maleic anhydride groups attached to it may form covalent bonds with the natural fibers. Previously, MAH-SEBS was also used as a compatibilizer to improve the adhesion in pineapple leaf fiber-reinforced SEBS composites [35,36].

To the best of our knowledge, PNF has not been used as reinforcement with the SEBS TPE matrix. Therefore, this study aims to utilize PNFs as reinforcement with the SEBS TPE matrix for a PNF-reinforced SEBS (PNF/SEBS) composite fabrication using the MAH-SEBS compatibilizer. The compatibilizer effect and fiber content effect were investigated on their adhesion and the mechanical and dynamic mechanical behaviors of the composites, which can be used for automobiles and other engineering applications.

## 2. Materials and Methods

### 2.1. Materials

The matrix polymer, styrene ethylene butylene styrene (SEBS) block copolymer, was obtained from BEC Materials (China) with a trading name of SEBS YH-510. SEBS YH-510 has over 70% vinyl content and 20 wt% styrene content with an MFI of 20 g/10 min (200 °C/5 kg) and a density of 0.89 gm/cm^3^. SEBS grafted with 1 wt% maleic anhydride, with a trading name of MAH-SEBS 901, was also obtained from BEC Materials (China) and was used as a compatibilizer in this study. The lignocellulosic pine needle fibers (PNFs) used as reinforcement in this study were obtained from pine needles. The fully dried fallen pine needles were collected from red pine trees. After washing and completely drying them, the dried pine needles were chopped and crushed to obtain the lignocellulosic pine needle fibers (PNFs) with a 0.5 mm average length. The PNFs were used without any further treatment.

### 2.2. Composite Preparation

**Figure 1:**
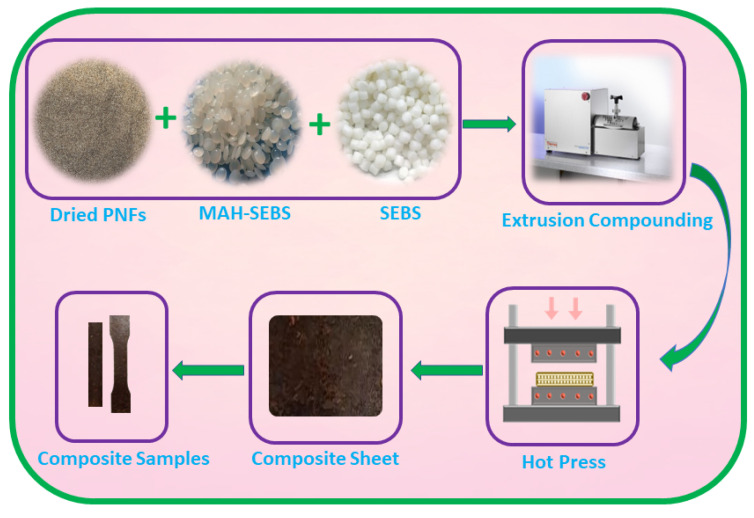
Composite preparation method.

To remove any present moisture on the raw materials, first, PNFs, SEBS, and MAH-SEBS were oven-dried overnight at 60 °C. The oven-dried reinforcing PNFs, matrix polymer SEBS, and MAH-SEBS compatibilizer were mixed manually by varying the PNF contents from 10 to 30 wt% with a fixed compatibilizer content of 5 wt%. The compositions of the constituents of the PNF/SEBS composites are recorded in Table 1. The mixtures of matrix polymer, reinforcing fibers, and compatibilizer were compounded using a twin screw compounder (HAAKE™ MiniCTW Micro-Conical, Thermo Fisher Scientific) using the temperature from 175 °C to 185 °C at a mixing speed of 140 rpm. Further, the compounded PNF/SEBS composites were hot pressed at 190 °C to obtain sheets of size 15 cm × 15 cm. Finally, according to the standard used, the composite samples were cut from the sheets for testing and characterization. In this study, the compatibilizer MAH-SEBS used to improve the fiber/matrix adhesion in the PNF/SEBS composite had a 99 wt% identical polymer backbone that is easily and completely miscible with the SEBS polymer matrix and has only 1 wt% maleic anhydride groups grafted to this identical backbone. Thus, with a 99% identical backbone, adding the MAH-SEBS into a neat SEBS polymer matrix may not exhibit any significant effect on the properties or may not change the material’s behavior. Therefore, the neat SEBS sample was considered as the reference sample for the comparison in this study. The composite preparation method is shown in Figure 1.

### 2.3. Chemical Interactions

An FTIR Spectrometer (Agilent Technologies Cary 630 FTIR, Republic of Korea) was used to record the FTIR spectra of each sample between 4000 cm^−1^ to 500 cm^−1^ using the pellet of the material and KBr powder mixture. Before recording the FTIR spectra, the materials were fully dried at 70 °C for 6 h using an oven.

### 2.4. Mechanical Testing

For the measurement of tensile properties, 5 specimens of each material were tested at ambient conditions at 25 °C temperature following the standard ISO 527-1:2019 on a universal testing machine (TO-100-IC, Test One, Republic of Korea), operated with a load and crosshead speed of 5 kN and 10 mm per min, respectively, and the average results of these specimens were recorded.

### 2.5. Morphological Analysis

For the morphological and interfacial adhesion analysis, the SEM pictures of broken specimens of the materials were captured by an FE-SEM (S-4000, Hitachi, Japan). Before capturing the images, the samples of the materials were coated with platinum using an ion sputtering machine.

### 2.6. Dynamic Mechanical Analysis

The materials’ dynamic mechanical characterizations were conducted by a DMA (Q800, TA Instrument, USA) on three-point bending following the ASTM D5023-15. The tests were performed between −120 °C and +120 °C at 3 °C per min heating rate at 1 Hz frequency and 10 μm amplitude.

### 2.7. Differential Scanning Calorimetry

The differential scanning calorimetry analysis of the materials was conducted by a DSC (200 F3 Maia, Netzsch, Germany) under an inert nitrogen atmosphere between the temperature range of −75 °C and +175 °C at a heating rate of 5 °C/min.

## 3. Results and Discussion

### 3.1. Chemical Interactions

**Figure 2:**
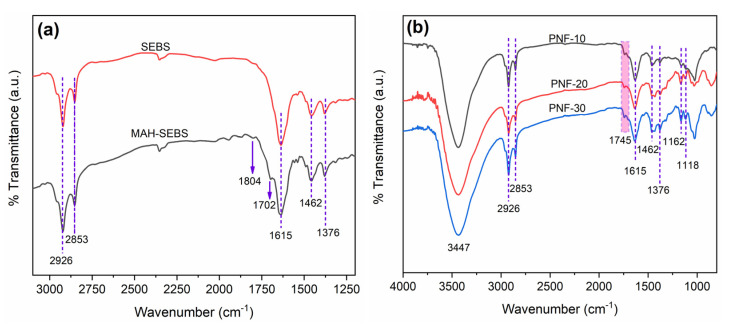
FTIR spectra of the (**a**) matrix, compatibilizer, and (**b**) composites.

**Figure 3:**
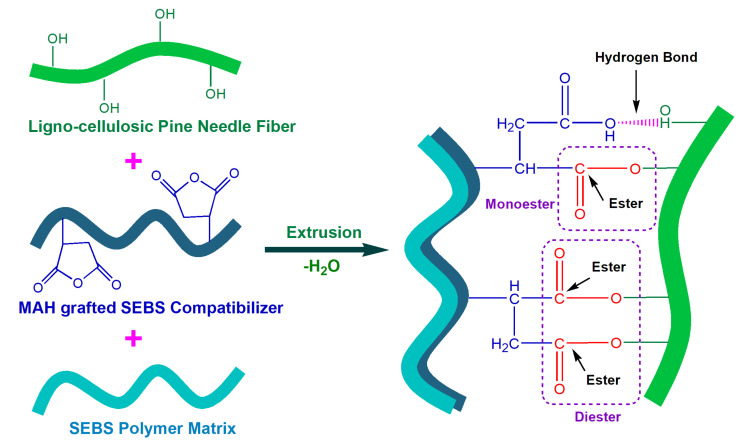
A possible scheme of ester formation between fiber, matrix, and compatibilizer.

The FTIR spectra of the SEBS polymer and the MAH-SEBS compatibilizer are recorded in Figure 2a. In the spectra of both SEBS and MAH-SEBS, the bands between 3000 to 2800 cm^−1^, at 2926 cm^−1^ and 2853 cm^−1^, designate the vibrations of the methylene of the aliphatics groups. The bands at 1462 cm^−1^ and 1376 cm^−1^ designate the bending vibrations of the CH_2_ and CH_3,_ and the band at 1615 cm^−1^ designates the C=C stretching of the aromatic ring of polystyrene of SEBS and MAH-SEBS. Two bands validate the presence of the MAH group in MAH-SEBS at 1804 cm^−1^ and 1702 cm^−1^ that do not exist in the SEBS spectrum. The 1702 cm^−1^ band shows the C=O asymmetric stretching of a cyclic anhydride of MAH-SEBS and confirms maleic anhydride grafting on the SEBS, whereas the 1804 cm^−1^ band shows the C=O of the maleic acid groups that may form by absorbing moisture from the environment [4]. The spectra of PNF/SEBS composites recorded in Figure 2b indicate that the band of C=O at 1702 cm^−1^ shows shifting to the higher wavenumber at 1745 cm^−1^. This band at 1745 cm^−1^ confirms the formation of strong ester bonds (–CO–O–) between the anhydride groups of the MAH-SEBS compatibilizer and the hydroxyl groups of the lignocellulosic PNFs. The SEBS backbone of the MAH-SEBS compatibilizer, identical and miscible, becomes completely mixed into the polymer matrix, and the functional MAH grafted onto its backbone reacts with the hydroxyl groups of the PNFs, forming strong covalent ester bonds. It indicates the strong interfacial adhesion between the PNFs and the SEBS (Figure 3).

Further, the intensity of the 1745 cm^−1^ band is also slightly increased with the fiber content, indicating that the ester bonds are more pronounced with increasing PNF contents. However, it has been noted that only one out of two possible acid groups can interact between MAH and the –OH of natural reinforcement [42] via a monoester, not as a diester. The modification itself is a monoester rather than a diester [43] and also via both the esters (mono and di), especially at temperatures over 100 °C [44]. Other studies indicate that ester formation increases while the reaction temperature rises, as the reaction temperature acts as a catalyst and possibly forms both monoesters and diesters [4,45,46]. The PNF/SEBS composites prepared in this research were compounded from 175 °C to 190 °C; thus, there is a possibility that both esters (mono and di) could develop between the –OH of PNF and MAH-SEBS (Figure 3). Moreover, the 3447 cm^−1^ spectral band shows that –OH bonds may also form between the –OH and MAH groups of the fiber and the MAH-SEBS, respectively (Figure 3), which also enhances the interfacial adhesion in the PNF/SEBS composites.

### 3.2. Mechanical Behaviour

**Figure 4:**
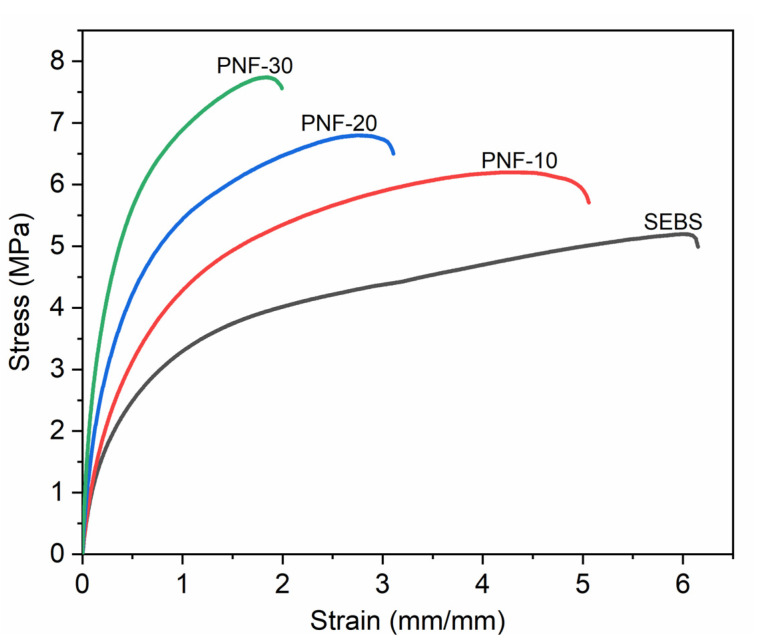
Tensile stress vs. strain curves of the matrix and the composites.

**Figure 5:**
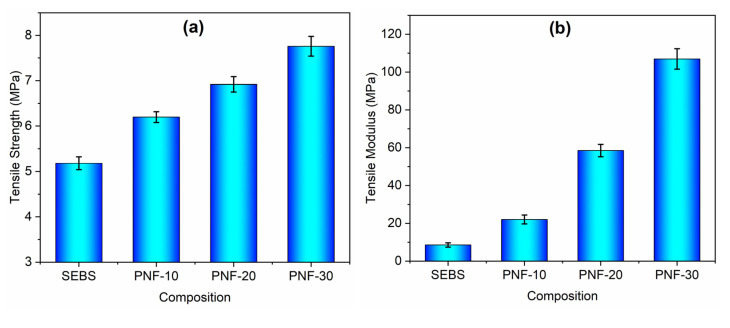

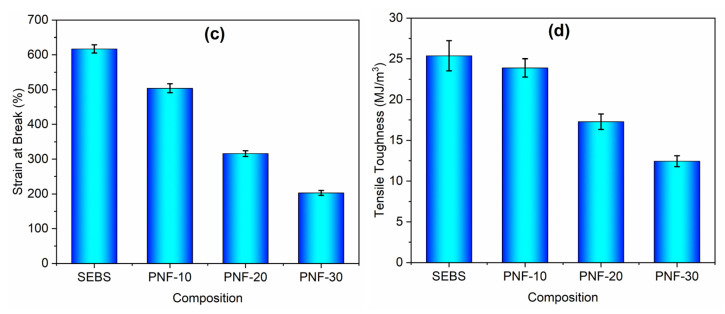
Tensile properties of the PNF/SEBS composites: (**a**) tensile strength, (**b**) tensile modulus, (**c**) tensile strain at break, and (**d**) tensile toughness.

The fiber content effect on the tensile behavior of the composites is recorded in Figure 4 as stress–strain curves of the composites, and Table 2 shows the tensile properties. Figure 4 clearly shows that with the increasing amount of reinforcing PNF in the matrix SEBS, the stress enhances to a greater extent while the strain is reduced. The neat SEBS polymer shows a tensile strength of 5.18 MPa and a tensile modulus of 8.56 MPa, while all the PNF/SEBS composites show higher strength and modulus than the neat polymer, indicating a maximum increase of about 50% in strength and a very high increase of about 1150% in modulus for the PNF−30 composition, relative to the matrix polymer (Figure 5a,b and Table 2). The PNF/SEBS composites show lower strain at break and toughness than the SEBS matrix, and all the composite compositions show an extension at break between 504.02 and 202.86% and toughness between 23.89 and 12.44 MJ/m^3^, indicating a maximum decrease of about 67% in the strain at break and a maximum reduction of about 51% in the toughness for the PNF-30 composition, relative to the matrix polymer (Figure 5c,d and Table 2). Adding brittle PNFs into the SEBS matrix reduces the ductility of the TPE matrix, resulting in the decreased strain at break and toughness of the composites. The PNF/SEBS composite with 30% PNF shows a 1150% improvement in the tensile modulus over 100 MPa, an exceptionally high mechanical property for thermoplastic materials. It is attributed to the strong interlocked network between the fibers and the matrix formed by the strong ester bonds between the PNFs and the SEBS matrix with the help of a compatibilizer, which possesses good interfacial adhesion between the fibers and matrix, offering excellent stress transfer from the reinforcing PNFs to the SEBS matrix.

### 3.3. Fractured Surface Morphology and Fiber/Matrix Interfacial Adhesion

**Figure 6:**
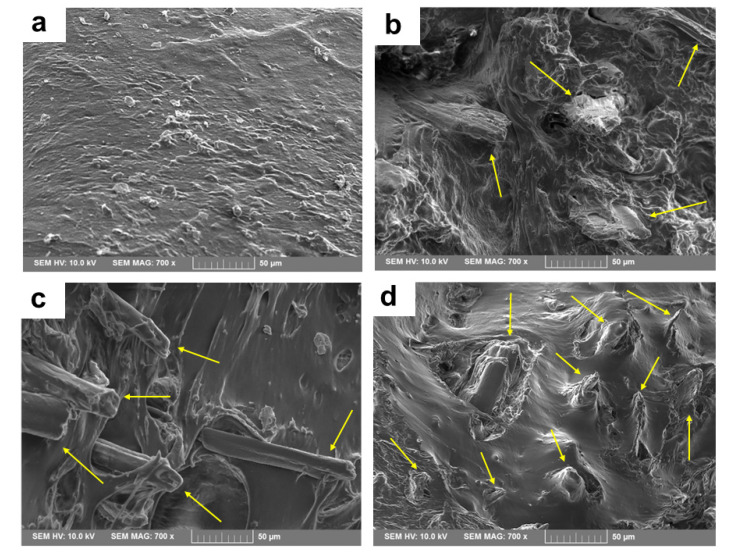
FE-SEM pictures of tensile fractured PNF/SEBS composites: (**a**) SEBS, (**b**) PNF-10, (**c**) PNF-20, and (**d**) PNF-30.

Figure 6 records the SEM pictures of the fractured surfaces of the PNF/SEBS composite specimens after the tensile test. The SEM image of the neat SEBS matrix (Figure 6a) exhibits a single-phase smooth surface without any filler or defects or flaws. The SEM pictures of each composite (Figure 6b–d) reveal that the SEBS matrix encases the reinforcing PNFs which are completely wrapped within the matrix: more fibers are shown for the composites with increasing reinforcing fiber contents. Moreover, irrespective of their fiber contents, the SEM pictures of all composites exhibit fiber breaking rather than fiber pull-out without any voids or gaps, representing the excellent fiber/matrix interfacial adhesion between the PNFs and the SEBS polymer with the MAH-SEBS compatibilizer. Furthermore, there is a chance that fibers may break during the extrusion compounding process of the composites. Hence, the final reinforcing fiber length in the composite for some fibers could be shorter than the initial length, 0.5 mm. However, it is challenging to determine the fiber length in the composites. As the composites’ tensile behavior improves with the fiber contents rising in the progressive composite compositions, the fibers in the composites have an effective length and aspect ratio.

### 3.4. Dynamic Mechanical Behaviour

The dynamic mechanical test for the SEBS and its composites was performed by setting the temperature range between −120 °C and +120 °C on a three-point bending mode. However, since the samples of the SEBS and its composites lost the grip of the support above 70 °C, the results of each material were recorded up to 70 °C.

**Figure 7:**
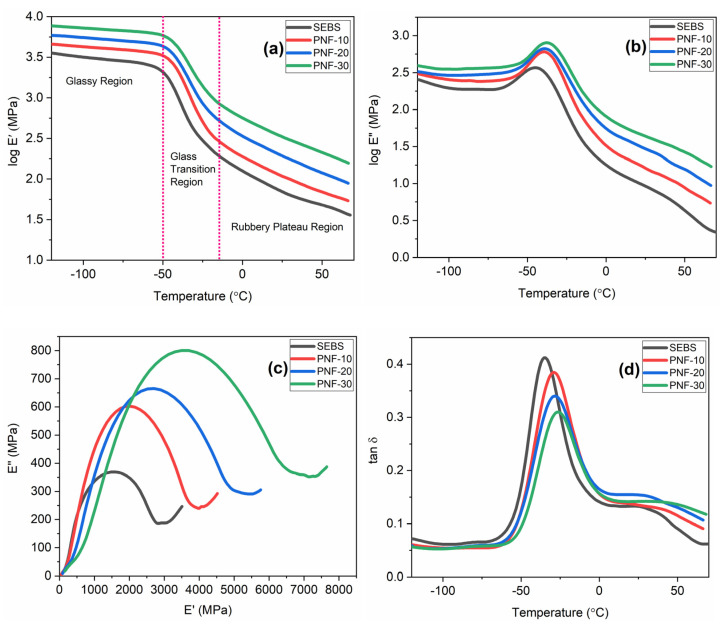
(**a**) Storage modulus, (**b**) loss modulus, (**c**) Cole–Cole plots, and (**d**) tan δ plots of the composites.

The storage modulus (E’) is a measurement of the energy stored in the material during a loading cycle, representing the stiffness of the material. The log E’ plots of the SEBS matrix and its composites with PNF contents are recorded in Figure 7a. Both the SEBS and their composites depict the glassy regions from −120 °C to −50 °C, the glass transition from −50 °C to −15 °C, and the rubbery plateau from −15 °C to 70 °C. The E’ plots of the SEBS and PNF/SEBS composites show the highest E’ in the glassy region and then decrease with increasing temperature in the glass transition and the rubbery plateau region. It can be explained by the following: In the glassy region, the materials’ macromolecular chains only exhibit vibrational and oscillational motions, giving them a high storage modulus; in the glass transition region, these chains gain energy for rotational movements, reducing the storage modulus; lastly, as the temperature rises over the glass transition region, the energy gained by these chains increases, causing the materials in the region of the rubbery plateau to lose even more of their capacity to store energy [47,48]. All the composite compositions have a higher storage modulus in each of the three regions compared to the matrix polymer, and when the fiber content increases, the storage modulus of the corresponding composites also increases. The stress transfer from the SEBS matrix to the PNFs becomes more pronounced, associated with the strong fiber/matrix adhesion caused by the strong ester bonds generated between the fibers and the matrix with the help of the compatibilizer. Similar outcomes were also seen with jute fibers reinforced with PP [49] and HDPE [50] and also with PS composites with cellulose fibers [51].

The loss modulus (E’’) measures energy dissipated as heat under deformation during a loading cycle. In NFRPCs, both the matrix and reinforcing natural fibers contribute to heat dissipation. The log loss modulus (log E’’) plots of the SEBS matrix and their composites with PNF are recorded in Figure 7b. Similar to the storage modulus, all the composite compositions exhibit larger loss moduli relative to the loss modulus of the matrix polymer in all three regions, and with increasingly higher fiber contents, the resulting composites exhibit higher loss moduli. It further suggests strong fiber/matrix adhesion in the composites increasing the dissipation of energy relative to those of the unreinforced matrix. Similar observations were also reported for cellulose fiber/PS [51] and jute fiber/HDPE [50] composites.

Cole–Cole plots of each material are recorded in Figure 7c. The curves for all the composite compositions are of an almost semi-circular arc shape and therefore indicate proper distribution and dispersion of the PNFs in the SEBS matrix of the PNF/SEBS composites. Cole–Cole plots [52] provide significant information about various systems, and modified versions of these plots were employed for polymer composites [53,54,55], where a semi-circular arc displays homogeneity whereas an uneven or irregular shape displays heterogeneity.

The tan δ plots of each material are recorded in Figure 7d. Generally, the temperature peaks in decreasing order are denoted as α and β peaks, indicating the glass transition temperature (T_g_) and secondary sub-glass transition temperature of a polymer, respectively; for copolymers that do not have a secondary glass transition temperature, these two peaks represent the T_g_ of the two components of the copolymers [56]. As for TPE, SEBS and its composite samples were recorded between −120 °C and 70 °C. It was challenging to record the peaks for the thermoplastic and elastomeric blocks, and only the peak for the elastomeric ethylene-butylene (EB) block was recorded. Accordingly, the peaks of tan δ vs. temperature plots (Figure 7d) of the matrix and the composites only indicate the T_g_ of the elastomeric blocks. The peak of the tan δ plot of the matrix polymer indicates the T_g_ at −35.13 °C for the elastomeric EB blocks of SEBS. The peaks of the tan δ plot of PNF-10, PNF-20, and PNF-30 composites indicate their T_g_ values at −29.52 °C, −27.64 °C, and −25.38 °C, respectively (Table 3) for elastomeric EB blocks. It is observed that adding PNF in the SEBS matrix shifts the T_g_ of the EB blocks of the composites to the higher temperature showing all the composites at significantly higher T_g_ values than that of the SEBS.

### 3.5. Differential Scanning Calorimetry

**Figure 8:**
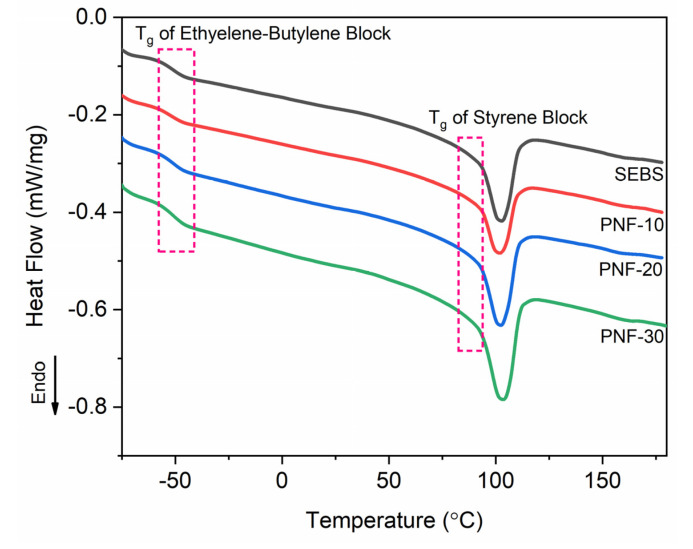
DSC thermograms of the composites.

Further, the DSC measurement was performed to investigate the effect of PNF on the Tg of the styrene block of the SEBS. In a DSC curve of a material, the endothermic change from the baseline represents the T_g_ of the material. The DSC thermograms of the SEBS matrix and its composites recorded in Figure 8 indicate two endothermic changes from their baseline, one between −55 °C and −45 °C and another between 95 °C and 85 °C, indicating the T_g_ of elastomeric EB block and thermoplastic styrene (S) blocks, respectively. For the elastomeric EB block, the SEBS matrix indicates the T_g_ at −54.30 °C, whereas the PNF−10, PNF-20, and PNF-30 composites indicate the T_g_ at −52.80 °C, −49.88 °C, and −47.25 °C, respectively (Table 3). For the thermoplastic S blocks, the SEBS matrix indicates the T_g_ at 88.74 °C, whereas the PNF-10, PNF-20, and PNF-30 composites indicate the T_g_ at 89.43 °C, 90.81 °C, and 91.73 °C, respectively (Table 3). Although the DSC measurement shows around 20 °C lower T_g_ than the DMA results for the EB block, similar to the DMA, all composites indicate a higher T_g_ for both the EB and S blocks than that of the SEBS, which further increases with the increasing the fiber contents. Relative to the SEBS matrix, the higher T_g_ of the composites and the T_g_ increase with the fiber content increasing from 10 to 30 wt% is attributed to the strong fiber/matrix adhesion generated by the strong ester bonds between the PNFs and the SEBS matrix with the help of a compatibilizer that reduces the mobility of the macromolecular chains in the glass transition.

Moreover, in addition to the mechanical and dynamic mechanical performance of the composite, the thermal degradation behavior and the water absorption behavior are also essential for any composite materials for their practical industrial application. Therefore, the thermal degradation behavior and water absorption behavior of the prepared PNF/SEBS composite may also be investigated in future work.

## 4. Conclusions

This study reported environmentally and economically advantageous pine needle fiber-reinforced styrene ethylene butylene styrene composites using an MAH-SEBS compatibilizer. The compatibilizer effect and the fiber content effect were investigated on their adhesion and on the mechanical and dynamic mechanical behaviors of the composites. Being identical, the SEBS backbone of the compatibilizer became completely mixed with the matrix, and the functional MAH groups grafted on this backbone formed strong covalent ester bonds, indicating the strong interfacial adhesion between the PNFs and SEBS as revealed by FTIR. This fiber/matrix adhesion leads to higher mechanical properties than the matrix, and these properties are further enhanced with the fiber contents in their composition as the composites with 30 wt% fiber content indicate a 1150% higher modulus and 50% higher strength than the matrix. Further, the SEM pictures confirmed this interfacial adhesion between the fiber and the matrix by indicating fiber breakage without any fiber pull-out and voids.

Moreover, the PNF/SEBS composites have better dynamic mechanical behavior than the matrix polymer, as indicated by a higher storage modulus, loss modulus, and glass transition temperature when compared to the polymer matrix that further improved with PNF content. Lastly, the strong interfacial adhesion generated through strong ester bonds, the greater enhancement in the mechanical properties, particularly, a tensile modulus of 107 MPa with a 1150% higher modulus than the matrix, and the excellent dynamic mechanical behavior of the prepared composites show the importance, significance, and premise of these composites as engineering materials potentially for automobiles and engineering applications.

## Figures and Tables

**Table 1:** Compositions of the PNF/SEBS composites.

Composition	Fiber (PNF, wt%)	Compatibilizer (MAH-SEBS, wt%)	Polymer (SEBS, wt%)
SEBS	00	00	100
PNF-10	10	05	85
PNF-20	20	05	75
PNF-30	30	05	65

**Table 2:** Tensile behavior of the materials.

Composition	Tensile Strength (MPa)	Tensile Modulus (MPa)	Strain at Break (%)	Toughness (MJ/m^3^)
SEBS	5.18 (±0.14)	8.56 (±1.1)	617.12 (±11)	25.38 (±1.8)
PNF-10	6.20 (±0.12)	22.03 (±2.3)	504.02 (±12)	23.89 (±1.1)
PNF-20	6.92 (±0.17)	58.48 (±3.2)	315.92 (±8)	17.28 (±0.9)
PNF-30	7.76 (±0.22)	106.92 (±5.4)	202.86 (±7)	12.44 (±0.6)

**Table 3:** The glass transition temperature (T_g_) of the PNF/SEBS composites.

Composition	T_g_ of EB Block by DMA (°C)	T_g_ of EB Block by DSC (°C)	T_g_ of S Block by DSC (°C)
SEBS	−35.13	−54.30	88.74
PNF-10	−29.52	−52.80	89.43
PNF-20	−27.64	−49.88	90.81
PNF-30	−25.38	−47.25	91.73

## Data Availability

The data presented in this study are available on request from the corresponding author.
